# 肺癌患者开胸术后发生肺不张的预防及治疗

**DOI:** 10.3779/j.issn.1009-3419.2010.03.09

**Published:** 2010-03-20

**Authors:** 永波 杨, 军 陈, 大兴 朱, 刚 陈, 志刚 李, 梅 李, 森 韦, 晓明 邱, 红林 赵, 懿 刘, 清华 周

**Affiliations:** 300052 天津，天津医科大学总医院，天津市肺癌研究所，天津医科大学总医院肺部肿瘤外科 Department of Lung Caner Surgery, Tianjin Lung Cancer Institute, Tianjin Medical University General Hospital, Tianjian 300052, China

**Keywords:** 肺癌, 开胸术, 术后肺不张, Lung Cancer, Thoracotomy, Postoperative atelectasis

## Abstract

**背景与目的:**

肺不张是开胸术后的常见并发症，严重时会危及患者生命。本文旨在分析和探讨肺癌患者行开胸术后发生肺不张的原因和围手术期的预防和处理措施，以便降低肺不张的发生率，并提高其治愈率，以进一步降低围手术期死亡率。

**方法:**

回顾性统计和分析我科因肺癌行开胸手术的374例患者中发生肺不张的资料和处理措施。

**结果:**

374例肺癌患者行开胸手术后发生肺不张的有14例，经积极有效地治疗后肺不张的肺叶均复张。

**结论:**

肺癌开胸术后肺不张发生率不高，有效的术前准备、良好的围术期处理和术后治疗可以降低开胸术后肺不张的发生率，提高治愈率。

## Abstract (en)

**Background and objective:**

Atelectasis is a common complication after thoracotomy, and it may threaten patients' life if it was not treated correctly and properly. The aim of this article is to explore and discuss the prevention and treatment for atelectasis during the perioperative period, and also to explore new methods for reducing the perioperative mortality due to atelectasis after thoracotomy.

**Methods:**

We retrospectively reviewed the medical records of 374 lung cancer patients who underwent thoracotomy in our department between Jan 2007 and Nov 2009.

**Results:**

Atelectasis occurred in 14 patients among all the 374 lung cancer patients who underwent thoracotomy. All the atelectasis returned to reexpansion after treatment.

**Conclusion:**

The incidence of atelectasis in these series is relatively low compared with the reports in literatures. Good perioperative preparation and perioperative treatment can remarkably decrease the incidence and mortality of atelectasis after thoracotomy in the treatment of lung cancer.

肺癌是发病率和死亡率增长最快的恶性肿瘤。对多数肺癌患者，尤其是早期患者，外科手术治疗仍然是重要和有效的治疗手段。肺不张是开胸手术尤其是肺叶切除术后的一个常见肺部并发症，如果没有及时诊断和治疗，会引起患者呼吸循环系统障碍，延长住院时间，严重时会危及患者生命。我科自2007年1月-2009年11月因肺癌行开胸肺叶切除术共374例，术后并发肺不张14例。本文总结本组肺癌患者术后并发肺不张的预防和术后处理措施。

## 1 临床资料

### 1.1 一般资料

全组手术患者共374例，所有病人均采用双腔导管气管内插管，静脉复合麻醉。其中男性258例，女性116例。60岁以上222例，60岁以下152例，平均年龄60.7岁。行右上叶切除术104例，右下叶切除术57例，右中叶切除术16例，右中上叶切除术12例，右中下叶切除术17例，左上叶切除术101例，左下叶切除术49例，左全肺切除术15例，其他手术方式3例。支气管袖式成型61例，隆突重建12例。术后发生肺不张的患者共14例，发病率为3.74%（14/374），其中男性12例，女性2例，60岁以上9例，60岁以下5例。平均年龄63.4岁，术前合并慢性支气管炎或肺部感染的8例，吸烟指数 > 400的1例，支气管袖式成型的2例。手术时间1 h-4 h，术后发生肺不张患者均有胸闷、咳嗽无力、不同程度的呼吸困难、气促、体温升高、心率增快、患侧呼吸音降低、发绀、纵隔向患侧偏移、血气分析提示低氧血症及血氧饱和度降低等临床表现，术后24 h-72 h依临床症状和影像学检查证实发生术侧或者健侧肺不张。

### 1.2 治疗方法

本组患者均行拍背排痰，加强体位引流，嘱患者做深呼吸、吹气球等锻炼，同时使用抗炎、解痉、雾化吸入、化痰、鼻导管吸痰、纤维支气管镜吸痰加冲洗等治疗。对一次纤维支气管镜吸痰后肺叶未复张的患者采取多次吸痰冲洗以促使肺叶复张。

## 2 结果

### 2.1 治疗效果

经过有效治疗后本组肺不张患者在2 h-48 h时内上述临床症状消失，血氧分压和氧饱和度均明显上升，经体格检查和复查胸片或胸部CT证实原不张的肺叶膨胀良好，复张率达到100%。

### 2.2 并发症及不良反应

无肺水肿、呼吸衰竭等并发症发生，4例患者出现肺部感染，经积极有效抗炎治疗后好转。在进行纤维支气管镜吸痰过程中2例出现不同程度的血氧饱和度下降、窦性心动过速，但能耐受，无大出血、严重心律失常、喉-支气管痉挛等严重并发症。其中1例患者进行了3次纤维支气管镜吸痰后肺叶才复张。

## 3 讨论

肺癌是全世界发病率和死亡率增长最快的恶性肿瘤^[1]^，在我国也是发病率和死亡率增长占首位的恶性肿瘤，肺癌男性死亡率最高为73.6/10万，女性为41.1/10万^[2]^。在肺癌的综合治疗中，外科手术治疗仍然是大多数肺癌患者尤其是早期肺癌患者最重要的根治性治疗手段。而肺不张是开胸手术尤其是肺叶切除术后的肺部常见并发症之一^[3]^，报道的发病率为2%-20%不等^[4-10]^。

肺不张的高危因素包括男性、高龄、吸烟、存在肺部感染、肺功能差^[11, 12]^、行右肺上叶切除术^[9]^等。其发生原因主要有：①气管插管气囊充气过多，使囊内压过大，压迫气管粘膜毛细血管，引起局部粘膜缺血。如时间过长，可导致局部粘膜充血、水肿，甚至坏死、脱落，丧失粘膜纤毛的波浪式排除分泌物运动功能。②由于切口疼痛，胸腔引流管的刺激，胸带包裹过紧限制了胸廓运动，患者体弱、呼吸肌无力导致支气管内气流速度下降而至通气量不足，在咳嗽时其剪力作用减弱，使气管内分泌物不易排出，导致肺不张。③术后过分限制液体的入量、吸入氧流量过大，均可导致痰液粘稠，不易咳出。④支气管成形术后，由于手术技巧不够熟练导致吻合口水肿、狭窄或成角畸形，均可导致支气管内分泌物潴留，引起肺不张等。肺癌患者开胸术后发生的肺不张是开胸术后肺部常见并发症之一，也是开胸术后导致死亡的原因之一^[13]^，根据肺不张发生的范围不同可以出现胸闷、气促、呼吸困难，也可有不同程度的咳嗽、咯血、喘鸣、发热等临床表现，体格检查可发现患者血压下降、心动过速，病变区叩诊浊音、呼吸音减弱，吸气时，如果有少量空气进入肺不张区，则可闻及干、湿啰音。上叶肺不张邻近气管，有时可闻及支气管呼吸音。一侧全肺不张可有患侧肋间隙变窄，气管及心脏向患侧移位。影像学表现有：①肺组织局部密度增高，呈均匀致密的毛玻璃状；②相应肺叶体积缩小；③叶、段肺不张一般呈钝三角形，宽而钝的面朝向胸膜面，尖端指向肺门，或呈扇形、三角形、带形、圆形等征象。当肺不张范围较大、其所造成的动静脉分流达到整个肺的20%时，将出现缺氧和紫绀，严重时患者将因为呼吸、循环衰竭而休克，甚至死亡。所以积极在开胸术后预防肺不张的发生及发生肺不张后进行有效地治疗有重要的意义。我们认为可以从以下几个方面来采取一些有效措施：

### 3.1 术前预防

术前一周训练病人深呼吸、锻炼腹式呼吸、有效咳嗽，吹气球以增加肺活量，进行上下楼梯训练以增加心肺功能和进行呼吸肌锻炼^[14]^，适当降低体重以增加胸廓顺应性，有呼吸道感染者应用抗生素控制呼吸系统炎症，加强雾化吸入，运用叩击背部、体位引流等方法，尽量促使肺部、支气管分泌物排除，支气管哮喘患者应该使用支气管解痉剂缓解支气管痉挛。要求吸烟病人应尽早戒烟，因为吸烟使小气道阻力增加，肺免疫功能降低。长期大量吸烟者，常伴有慢性支气管炎和肺气肿等，均可影响通气功能，严重时可极大地阻碍气体交换，戒烟后呼吸道纤毛粘液转运系统功能会改善，患者的血氧运输能力将增强，因此须绝对戒烟1月以上再进行手术，因为资料显示吸烟所致肺损害会随时间的延长及吸烟量的增加而加重，其发生肺不张的几率也明显增加^[15, 16]^。

### 3.2 术中预防

选择合适的气管插管，套囊充气要适量，囊内压不要超过4.0 kPa（30 mmHg）^[8]^，定时作短时间放气，以恢复局部气管粘膜血液供应，如可能应选用大容量低压套囊。尽可能缩短手术时间，术中定时行气管内吸痰和膨肺；术后应在患者清醒、自主呼吸恢复良好后拔出气管插管，且拔管前要彻底吸净呼吸道内分泌物。术中避免对肺组织进行挤压，以免导致肺表面活性物质减少而使肺顺应性下降，对于支气管成形术病例，术中要设计好吻合口，以防成角畸形，吻合时可以采用粘膜外缝合法，可减轻吻合口水肿，避免吻合口肉芽形成。

### 3.3 术后处理

体位：术后麻醉未清醒前，去枕平卧6 h，头偏向一侧，及时吸出呼吸道内分泌物及呕吐物，以防呼吸道阻塞，保持呼吸道通畅。全麻清醒后可以采取半坐位以利于胸液的引流和患者进行有效地深呼吸。同时应该避免胸带包裹过紧而限制了胸廓的运动。

有效止痛：采用硬膜外置管、自控式镇痛泵或者吗啡等止痛药物以提高患者咳嗽时对胸部切口疼痛的耐受力，消除其对咳嗽时切口疼痛的恐惧感。有效的镇痛可以降低术后肺部并发症的发生，减少住院时间^[17]^。

主动排痰：患者清醒后鼓励其进行有效的大而有力咳嗽及做深呼吸。患者进行咳嗽时，医务人员可以用双手按压患者胸廓，吸气时双手放松，咳痰时双手加压保护胸部切口在咳嗽时不会因为震荡而引起疼痛，并按压胸骨柄切迹处气管，促发患者有效咳嗽。对于痰液黏稠不易咳出时可采用雾化吸入以润滑气道，稀释分泌物，再配合有效地拍背，以利痰液的排除。甚至可以进行环甲膜穿刺气管内滴药，诱发强烈有效咳嗽，促进排痰。

被动排痰：若是痰液比较黏稠，位置较深，可辅以经鼻或口腔的鼻导管吸痰，刺激气管粘膜，引起较强烈的反射性咳嗽或直接吸出痰液，以排除堵塞支气管的痰液，解除气道堵塞。若痰量持续过多，且患者咳嗽无力，排痰无效，或者支气内有血凝块、痰栓堵塞管腔患者无法自行咳出时，可以应用纤维支气管镜反复吸痰，以吸除气道内的分泌物及痰块，若血痂干结、痰液粘稠，可以向支气管内注入适量灌洗液进行灌洗，以稀释分泌物及冲洗气道。而对于充血水肿的气道黏膜可局部滴入地塞米松，高浓度激素对局部有消炎、抗过敏、解除支气管痉挛等作用，利于减轻气道狭窄。纤支镜可以直视支气管内的情况，证实胸片所提示的肺不张部位，吸尽痰液及其它阻塞物，并可进行支气管肺泡灌洗、支气管内局部给药和获取下呼吸道分泌物进行细菌培养及药敏试验，从而迅速改善支气管的引流和通气，有助于肺复张和提高对肺部感染的控制率，是治疗术后肺不张较为安全有效的方法。因为手术本身和肺不张均对呼吸功能有影响，而纤支镜的插入又阻塞了部分气道，并有可能激发支气管痉挛加重缺氧，还可能使患者发生大出血、窒息、喉头水肿、心律失常、心脏骤停甚至医源性异物残留^[17]^等严重并发症，所以要求术者应该具备相当的临床经验，在操作过程中如果患者出现心律紊乱、血氧降低时，应立即停止操作，并充分给氧，待血氧饱和度恢复后再行操作。同时在进行吸引时，应避免压力过大，不宜持续吸引，可采取间歇吸引方式，以避免黏膜损伤、水肿^[19]^。另外也可以通过支气管镜使用表面活性物质以减少肺不张的复发^[20]^。对于1次气管镜吸痰治疗后肺叶未能复张的患者，可以多次进行气管镜吸痰和灌洗。在本组患者中，有1例患者经过了3次支气管吸痰治疗后肺叶才复张。

另外值得注意的是，术式的选择对于开胸术后肺不张的发生率也有显著影响，在有条件的单位，选择胸腔镜辅助肺叶切除术相比开胸肺叶切除术能有效降低术后肺不张等肺部并发症的发生率^[21]^。

总之，肺不张是开胸术后的常见并发症，如果未能及时发现且未给予准确的治疗，严重时将会危及患者生命。我们在围手术期采取了积极有效的预防和治疗措施，显著降低了肺不张的发生率，并有效提高了其治愈率。
